# Effectiveness of Surgical Excision in Preventing Recurrence of Multilocular Brain Abscess

**DOI:** 10.7759/cureus.80105

**Published:** 2025-03-05

**Authors:** Keijiro Murakami, Tomoki Kidani, Shin Nakajima, Yonehiro Kanemura, Tomohiko Ozaki, Katsunori Asai, Nobuyuki Izutsu, Koji Kobayashi, Yosuke Fujimi, Toshiyuki Fujinaka

**Affiliations:** 1 Department of Neurosurgery, Osaka National Hospital, Osaka, JPN; 2 Department of Biomedical Research and Innovation, Institute for Clinical Research, Osaka National Hospital, Osaka, JPN; 3 Department of Neurosurgery, Osaka University Graduate School of Medicine, Suita, JPN

**Keywords:** brain abscess, brain abscess excision, intra-ventricular rupture of brain abscess, multiocular cyst, . surgical excision

## Abstract

Background

Despite advances in surgical treatment and antimicrobial therapy reducing mortality and recurrence rates of brain abscesses, many patients still face a challenging clinical course. To assess whether selecting an appropriate surgical approach influences patient outcomes, we retrospectively analyzed cases at our hospital, with a particular focus on abscess morphology.

Methods

We retrospectively analyzed 20 patients who underwent surgery for brain abscesses at our hospital between March 2005 and April 2022. Brain abscesses were classified as either simple or multilocular. Recurrence was defined as an increase in abscess size on postoperative contrast-enhanced imaging, while a poor outcome was defined as a >1-point increase in the modified Rankin Scale score at discharge.

Results

Of the 20 patients, 18 were included in the analysis. The mean patient age was 67 ± 15 years, and 17 were men. Among the 18 abscesses, 16 were classified as simple and two as multilocular. A total of 22 surgeries were performed, including 18 as initial treatments, three for recurrence, and one for a second recurrence. The procedures consisted of two excisions, 14 aspirations, five external ventricular drainage (EVD) procedures, and one aspiration combined with EVD. In cases treated with aspiration, the recurrence rate was significantly higher in the multilocular abscess group than in the simple abscess group (75% vs. 9.1%), with an OR of 24.0 (95% CI, 1.26-63.82; p = 0.011).

Conclusions

In patients with multilocular brain abscesses, our findings suggest that aspiration is associated with a higher recurrence rate, while surgical excision may be a more effective treatment approach. However, further studies are needed to confirm this benefit.

## Introduction

Brain abscess is a rare but serious condition, with an incidence in the United States ranging from 0.3 to 1.3 cases per 100,000 people per year. Similar rates have been reported in other developed countries [1]. Despite advances in surgical techniques and antimicrobial therapies, many patients face a challenging clinical course [1,2]. These difficulties highlight the need to refine treatment strategies to enhance patient outcomes.

The main surgical approaches for managing brain abscesses are aspiration and surgical excision, each with distinct advantages and limitations depending on the abscess’s size, location, and morphology. In cases complicated by intraventricular rupture, external ventricular drainage (EVD) is typically required to address secondary complications. Treatment decisions should be made on a case-by-case basis [3].

Multilocular abscesses are believed to carry a higher risk of recurrence and poorer neurological outcomes [4]. This study aims to identify the most effective surgical techniques for managing multilocular brain abscesses to improve patient prognosis.

## Materials and methods

This study was approved by the ethics committee of our institution and conducted in accordance with its guidelines (approval number 23019). All eligible patients were informed about the study through an opt-out notice posted on the ethics committee’s website.

We retrospectively reviewed 20 patients who underwent surgery for brain abscesses at our hospital between March 2005 and April 2022. Cases of brain abscesses resulting from head trauma or craniotomy were excluded. Additionally, a patient with a tuberculous brain abscess was excluded due to the distinct nature of its treatment, as well as one patient with multiple brain abscesses.

The following data were collected: age, sex, medical history, number and location of brain abscesses, abscess size and volume, presence of multilocularity, presence of intraventricular rupture of brain abscess (IVROBA), prior infection, antibacterial agents used, surgical technique, recurrence, reoperation, causative organisms identified in abscess cultures, and modified Rankin Scale (mRS) scores before onset and at discharge.

**Figure 1:**
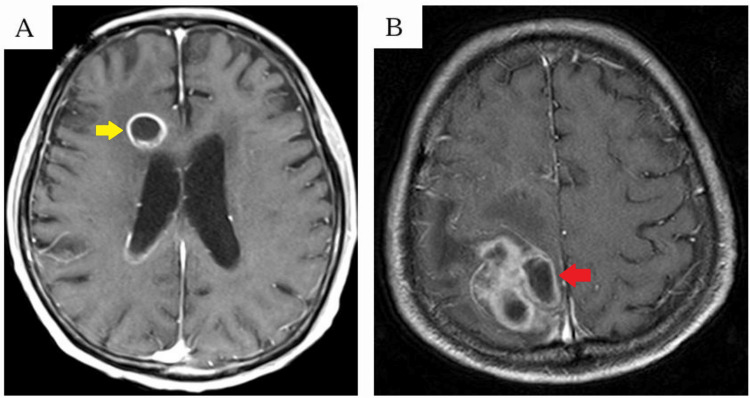
Abscess classification (A) Yellow arrows indicate a simple abscess. (B) Red arrows indicate a multilocular abscess.

Abscesses were classified as either simple or multilocular based on contrast-enhanced MRI or CT. Those displaying ring enhancement of only the outer wall (Figure 1A) were categorized as simple, while those with enhancing internal septa were classified as multilocular (Figure 1B).

Abscess volume was estimated using the following equation:

\[ \frac{4}{3} \pi \times \frac{\text{length}}{2} \times \frac{\text{width}}{2} \times \frac{\text{height}}{2} \]

The surgical techniques employed included aspiration, excision, and EVD for cases of IVROBA. All excisions were performed under general anesthesia using an appropriately sized craniotomy. Aspiration and EVD procedures were conducted under either general or local anesthesia, utilizing a single burr hole or a small craniotomy. Intraoperative image guidance with a surgical navigation system and/or ultrasonography was used as needed.

Recurrence was defined as an increase in abscess size on postoperative contrast-enhanced MRI or CT. A poor outcome was defined as an increase of more than one point in the mRS score at discharge.

Treatment strategy

As part of our current institutional policy, surgical treatment is considered for brain abscesses that are surgically accessible and meet a size threshold of approximately ≥2 cm. This approach facilitates the identification of causative microorganisms and the selection of appropriate antimicrobial therapy. Retrospectively, surgeries have been performed based on this criterion.

In recent years, collaboration with the Department of Infectious Disease and the Infection Control Team has contributed to a more standardized approach to antibiotic selection. This criterion aligns with the Guidelines for the Diagnosis and Treatment of Brain Abscess, published by the European Society of Clinical Microbiology and Infectious Diseases (ESCMID) in 2024.

Statistical analysis

Differences in continuous variables were assessed using the two-sided Student’s t-test, while categorical variables were compared using the chi-square test or Fisher’s exact test. A p-value of <0.05 was considered statistically significant.

Statistical analyses, including sample power calculations, were performed using JMP Pro 17 software (SAS Institute Inc., Cary, NC, USA) to evaluate the adequacy of the study’s statistical power. A statistical power of 0.8 (80%) or higher was considered sufficient to detect a true effect.

## Results

Patient and abscess characteristics

Over a 17-year period, our institution treated 20 cases of brain abscesses. After excluding one case of tuberculous brain abscess and one case of multiple brain abscesses, a total of 18 cases were included in the analysis.

The mean patient age was 67 ± 15.0 years, and 17 patients (94.4%) were men. Four patients had a history of cancer, including leukemia, lung cancer, esophageal cancer, and stomach cancer. Another four patients had a documented prior infection, such as vertebral osteomyelitis after radiotherapy, a pressure ulcer of the buttocks, a leg muscle abscess, or sinusitis.

IVROBA was present in six patients before the initial surgery, while one patient developed IVROBA at the time of recurrence. At diagnosis, 13 patients (72.2%) had an mRS score between 0 and 2. By discharge, only five patients (27.8%) maintained an mRS score within this range (Table 1).

**Table 1:** Patient characteristics Values are presented as N (%). IVROBA, intraventricular rupture of brain abscess; mRS, modified Rankin scale

Variable	N = 18
Age (years)	67 ± 15 (mean ± SD)
Sex	
Male	17 (94.4%)
Female	1 (5.6%)
Comorbidities	
Malignant disease	4 (22.2%)
Diabetes mellitus	3 (16.7%)
Prior infection	4 (22.2%)
IVROBA	7 (38.9%)
Pre-onset mRS score	
0-2	13 (72.2%)
3-5	5 (27.8%)
mRS at discharge	
0-2	5 (27.8%)
3-5	9 (50.0%)
6	4 (22.2%)

Among the 18 abscesses, 16 were simple, and two were multilocular. The most common location was the frontal lobe (11 cases), followed by the parietal lobe (one case), occipital lobe (two cases), cerebellum (two cases), temporal lobe (one case), and thalamus (one case).

Three abscesses recurred and required additional treatment; of these, one was simple, and two were multilocular. Among abscesses with available measurements, including both initial and recurrent cases, the mean abscess length was 3.63 ± 1.36 cm, and the mean volume was 1.85 ± 16.6 cm³ (Table 2).

**Table 2:** Characteristics of brain abscesses and surgeries ^*^ Excluding three cases for which accurate measurements were unavailable ^†^ Among the 22 cases, two involved the detection of two different bacterial species. Values are presented as N (%). EVD, external ventricular drainage

Variable	N = 22
Surgical procedure	
Aspiration	14 (60.0%)
Initial	12
Recurrence	2
Excision	2 (16.0%)
Initial	1
Second recurrence	1
EVD	5 (20.0%)
Initial	4
Recurrence	1
Aspiration and EVD	1
Shape	
Simple	17 (77.3%)
Initial	16
Recurrence	1
Multilocular	5 (22.7%)
Initial	2
Recurrence	2
Second recurrence	1
Abscess measurements	
Long Diameter (cm, mean ± SD)	3.63 ± 1.36^*^
Volume (cm³, median ± SD)	18.5 ± 16.6^*^
Location	
Frontal lobe	12 (54.5%)
Initial	11
Recurrence	1
Parietal lobe	3 (13.6%)
Initial	1
Recurrence	1
Second recurrence	1
Occipital lobe	3 (13.6%)
Initial	2
Recurrence	1
Temporal lobe	1 (4.5%)
Cerebellum	2 (9.0%)
Thalamus	1 (4.5%)
Pathogenic bacteria (N = 24)^†^	
* Streptococcus*	9 (37.5%)
* Peptostreptococcus*	2 (8.3%)
* Fusobacterium*	2 (8.3%)
* Haemophilus*	1 (4.2%)
* Corynebacterium*	1 (4.2%)
* Enterococcus*	1 (4.2%)
* Porphyromonas*	1 (4.2%)
Culture negative	7 (29.2%)

Surgical information

A total of 22 surgical procedures were performed on 18 patients for 18 brain abscesses. Anesthesia records prior to 2006 were unavailable due to data deletion. Among the 19 procedures performed since then, 16 were conducted under general anesthesia, while three were performed under local anesthesia (two aspirations and one EVD procedure).

An abscess cavity drain was placed in 16 procedures. Intraoperative ultrasonography was used in eight procedures, while surgical navigation was utilized in nine procedures, with its use being documented since 2012. Among the 11 patients who underwent aspiration as the initial surgery, eight had a drain placed. The average duration of drain placement was 5.1 ± 3.8 days.

Postoperative contrast-enhanced MRI or CT was used for follow-up in 11 procedures (50%), with all recurrent cases being monitored using contrast-enhanced imaging. Complete abscess removal was observed in three cases (two aspirations and one excision), none of which experienced recurrence. Among the remaining eight cases, three abscesses recurred.

Four cases, all classified as mRS 6, were followed up using non-contrast CT, while another four were monitored with non-contrast MRI. The mean follow-up duration, calculated from the last surgery to the last follow-up, was 773.4 ± 1,512 days (range: 1-6,505 days).

Pathogenic bacteria and antibiotics

A causative bacterial species was identified in 15 of 22 surgical procedures through abscess cultures. The most commonly isolated bacteria were Streptococcus (nine cases), followed by *Peptostreptococcus *(two cases), *Fusobacterium *(two cases), *Haemophilus *(one case), *Corynebacterium *(one case), *Enterococcus *(one case), and *Porphyromonas* (one case). Coinfection with two bacterial species was detected in two patients.

All patients received optimal antimicrobial therapy based on antimicrobial susceptibility testing. When culture results were negative, empirical antibiotic treatment was administered. If cultures were positive, antibiotic therapy was adjusted to a more targeted regimen based on susceptibility testing.

A change in the duration of antibiotic treatment was observed before and after 2011. Prior to 2011, patients continued oral antibiotics after discharge, with most receiving treatment for over 12 weeks (maximum duration: 19 weeks and four days). After 2011, the median duration of antibiotic treatment was 48 days.

Treatment and recurrence

A total of 18 initial surgical procedures were performed for 18 brain abscesses in 18 patients. Among the 16 simple abscesses, treatments included 10 aspirations, one excision, four EVD procedures, and one aspiration combined with EVD. Both multilocular abscesses were initially treated with aspiration.

**Figure 2:**
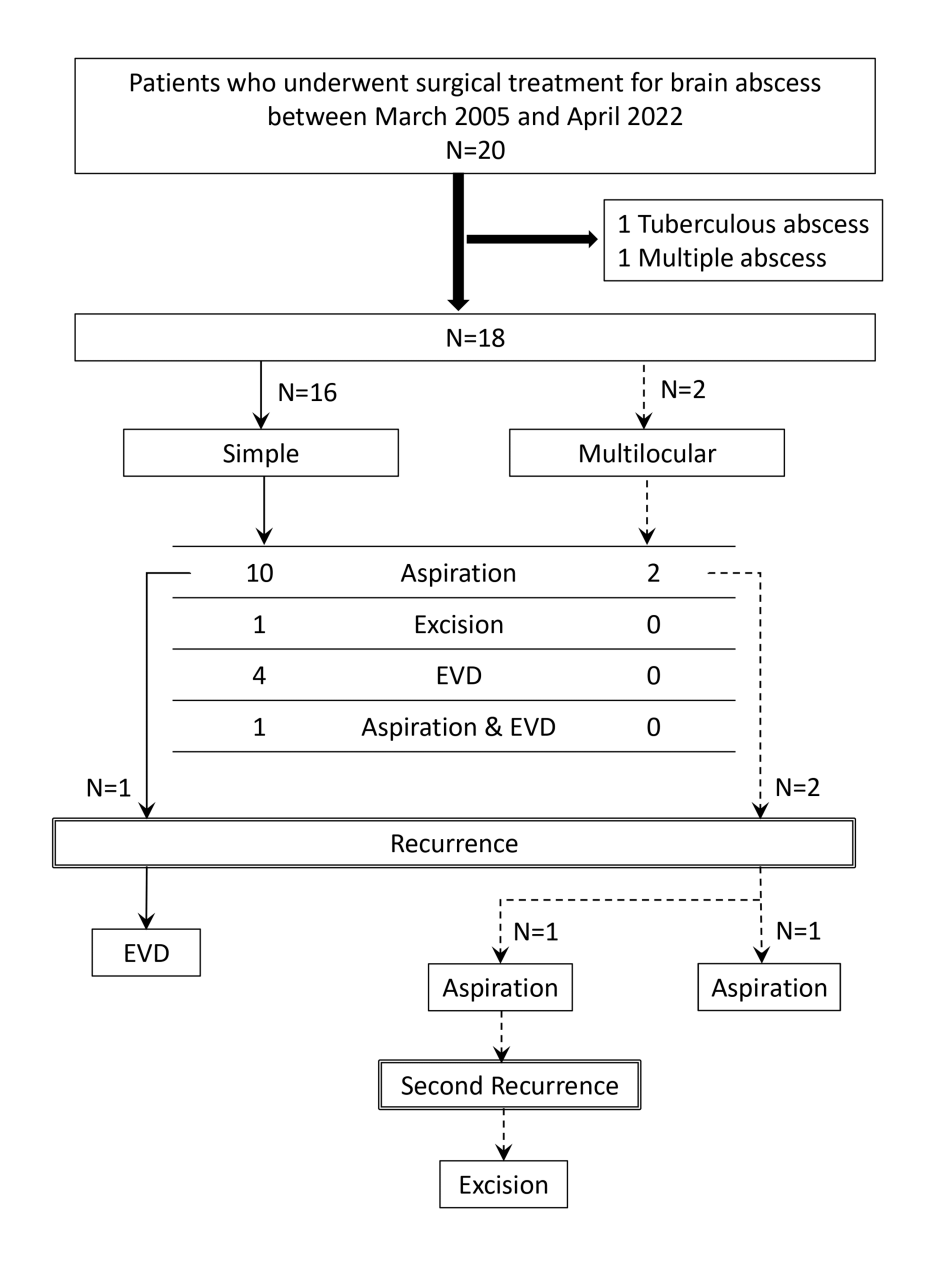
Treatment and recurrence EVD, external ventricular drainage

Overall, three abscesses recurred following initial treatment: one simple and two multilocular. All had been initially treated with aspiration and required reoperation. Among the two recurrent multilocular abscesses, one was managed with repeat aspiration, while the other underwent excision. The recurrent simple abscess ruptured into the ventricle and was treated with EVD. One multilocular abscess recurred a second time after re-aspiration and was ultimately treated with excision (Figure 2).

All recurrences occurred following aspiration. Among the 14 abscesses treated with aspiration (12 initial and two recurrent), four recurred (28.6%). No recurrences were observed after other surgical procedures. However, the recurrence rate did not significantly differ between procedures.

Mean abscess length and volume showed no significant differences between the simple and multilocular abscess groups. Among abscesses treated with excision, recurrence rates did not differ significantly between the groups (p = 1). However, in cases treated with aspiration, the recurrence rate was significantly higher in the multilocular group (75% vs. 9.1%), with an OR of 30.0 (95% CI: 1.26-63.82, p = 0.011) (Table 3).

**Table 3:** Comparison between multilocular and simple brain abscess groups ^*^ p < 0.05: considered statistically significant ^†^ Excluding three cases for which accurate measurements were unavailable

Parameter	Multilocular (N = 5)	Simple (N = 17)	p-Value	OR	95% CI
Long diameter (cm), mean ± SD	4.18 ± 1.4	3.43 ± 1.3^†^	0.37	-	-
Volume (cm³), mean ± SD	27.1 ± 19.1	15.4 ± 14.4^†^	0.306	-	-
Recurrence, N (%)	3 (60%)	1 (5.9%)	0.0239^*^	24	1.62-352.1
Recurrence after aspiration, N (%)	3/4 (75%)	1/11 (9.1%)	0.011^*^	30	1.26-63.82
Recurrence after excision, N (%)	0/1 (0%)	0/1 (0%)	-	-	-

Outcomes and associated factors

At the time of abscess diagnosis, the mRS scores were as follows: 0 in nine patients, 1 in one patient, 2 in three patients, 3 in four patients, and 4 in one patient. At discharge, mRS scores were 0 in three patients, 1 in two, 3 in five, 4 in one, 5 in three, and 6 in four.

A poor outcome, defined as an increase of more than one point in mRS score at discharge, was observed in 13 patients (72.2%). Four patients (22.2%) died, two due to postoperative cerebral hemorrhage and two due to pneumonia.

**Figure 3:**
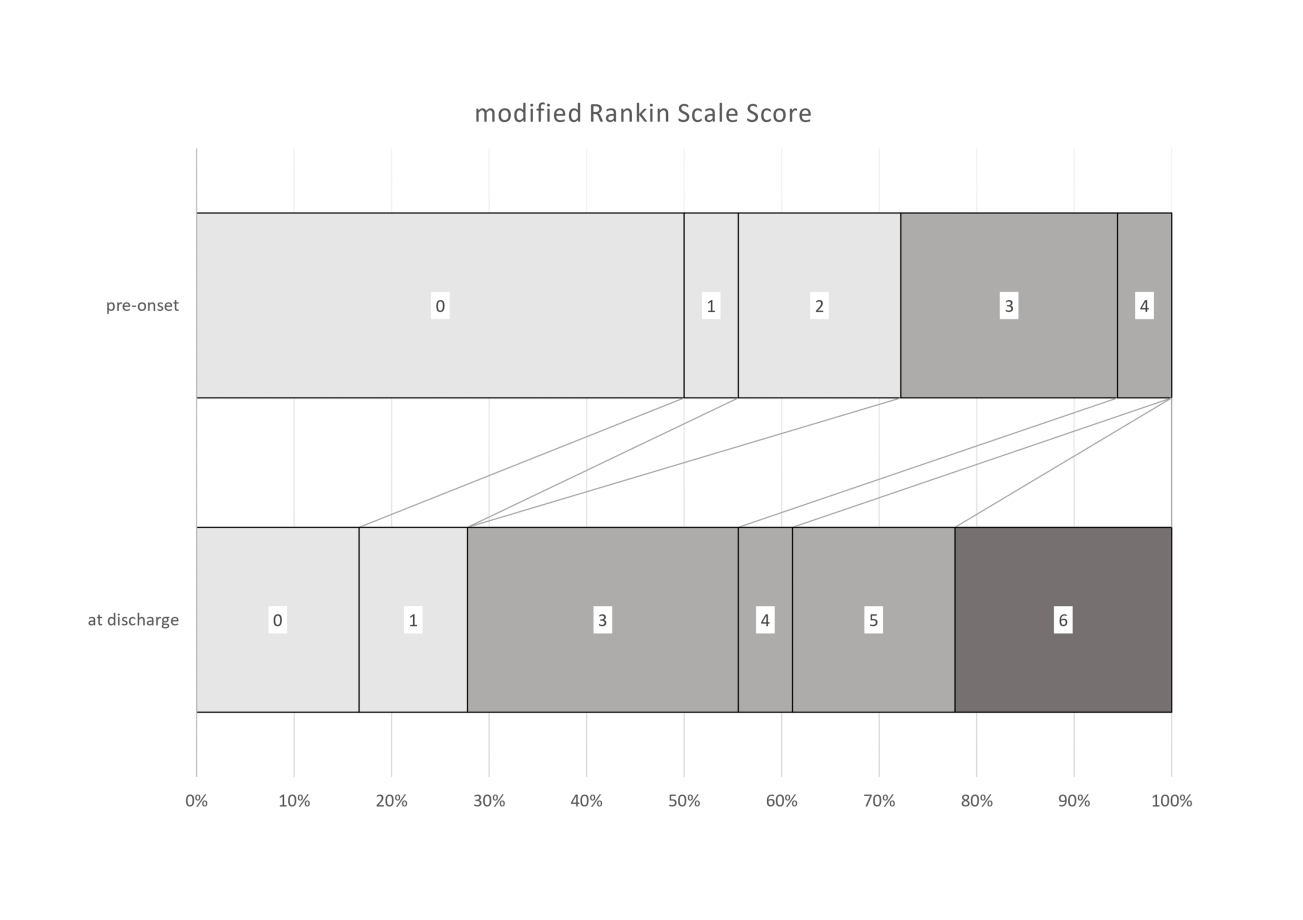
mRS score mRS, modified Rankin Scale

No significant differences were found between the poor and good outcome groups in terms of age, multilocularity, IVROBA, surgical procedure, mRS score at diagnosis, or recurrence (Figure 3, Table 4). The statistical power for all analyses was below 0.8.

**Table 4:** Outcomes and associated factors p < 0.05: considered statistically significant A statistical power of 0.8 or higher is considered sufficient to detect meaningful differences. IVROBA, intraventricular rupture of brain abscess; EVD, external ventricular drainage; mRS, modified Rankin Scale

Prognosis	Good (N = 5)	Poor (N = 13)	p-Value	Statistical power
Age: mean (years)	62.6	68.5	0.578	-
Multilocular abscess	0	2	1.0	0.769
IVROBA	1	6	0.596	0.208
Final surgical procedure				
Aspiration	4	7	0.2939	-
Excision	0	2		
EVD	0	5		
Pre-onset mRS ≤2	3	10	0.583	0.105
Recurrence	0	3	0.522	0.782

Representative case

A 64-year-old man was admitted to our hospital with left leg pain and swelling. He was initially diagnosed with cellulitis and started on intravenous cefazolin (3 g/day). After an intramuscular abscess was detected in the affected leg, his antimicrobial regimen was switched to intravenous sulbactam/ampicillin (12 g/day) and clindamycin (1,800 mg/day). He underwent operative debridement under general anesthesia but subsequently developed seizures and left hemiplegia.

**Figure 4:**
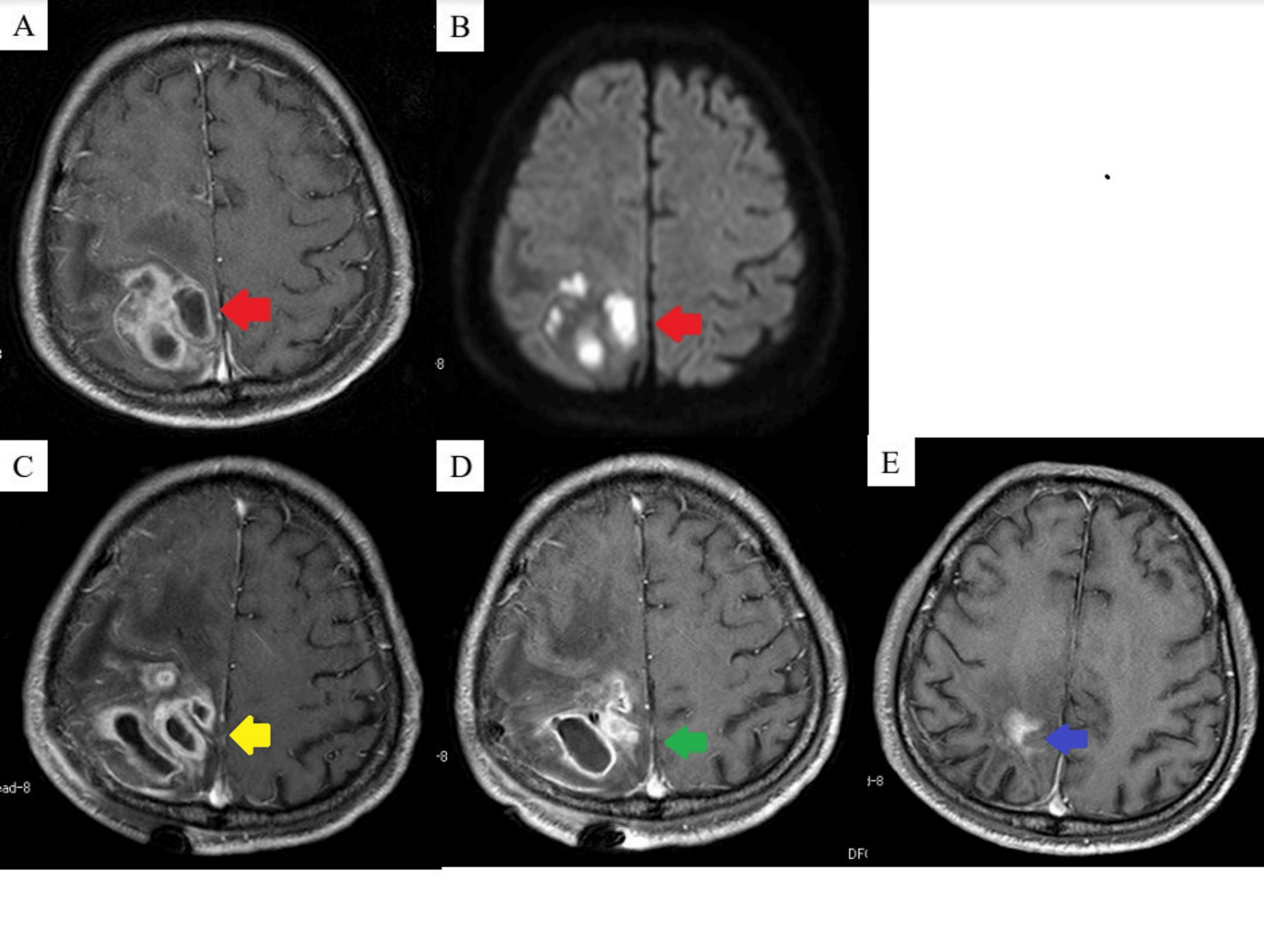
Imaging findings of the representative case (A) MRI T1CE: The red arrow indicates the preoperative abscess. (B) MRI DWI: The red arrow indicates the preoperative abscess. (C) MRI T1CE: The yellow arrow indicates the abscess at first recurrence. (D) MRI T1CE: The green arrow indicates the abscess at the second recurrence. (E) MRI T1CE: Follow-up MRI shows no recurrence (blue arrow). DWI, diffusion-weighted imaging; T1CE, T1-weighted contrast-enhanced MRI

Contrast-enhanced MRI revealed a multilocular high-intensity mass with ring enhancement in the right occipital lobe (Figure 4A, 4B), leading to a diagnosis of brain abscess. His antimicrobial treatment was adjusted to ceftriaxone (4 g/day), vancomycin (3 g/day), and metronidazole (1,500 mg/day). Despite this, a follow-up MRI showed an increase in abscess size, necessitating aspiration under general anesthesia.

Postoperative MRI confirmed recurrence (Figure 4C), prompting a second aspiration 13 days after the initial procedure. Due to a second recurrence 24 days later, a total excision was performed (Figure 4D). Follow-up MRI showed no further recurrence (Figure 4E). No causative organism was identified in any of the abscess cultures. Antimicrobial therapy was continued for eight weeks postoperatively. The patient had an mRS score of 3 at discharge.

## Discussion

The incidence of brain abscesses in developed countries has been reported to range from 0.3 to 1.3 cases per 100,000 people per year. Chuo Ward in Osaka, where our hospital is located, has a population of approximately 107,000. Given this, the 20 cases observed over 17 years align with these reported incidence rates [1].

Medical treatment of brain abscess

Brain abscesses can develop through contiguous, hematogenous, posttraumatic, or postoperative infection routes. Contiguous infections originate from nearby sources such as otitis media, mastoiditis, sinusitis, or meningitis. Dental infections may spread to the brain through both contiguous and hematogenous routes, while hematogenous abscesses often stem from infections in the lungs or heart [2,5]. Delayed initiation of antimicrobial therapy is linked to poor outcomes, highlighting the importance of early empiric treatment. If a specific pathogen is identified, antibiotic therapy should be adjusted based on susceptibility testing [6,7].

*Streptococci *are the most frequently reported causative organisms in brain abscesses [1] and are also commonly associated with otorhinolaryngologic infections [8]. In our study, one patient had sinusitis prior to developing a brain abscess. Anaerobic bacteria such as *Porphyromonas*, *Fusobacterium*, and *Peptostreptococcus* - all oral commensals linked to dental caries and periodontal disease [9] - can act as opportunistic pathogens in immunocompromised individuals and have been increasingly recognized as brain abscess causative agents since the 1900s [5]. Fusobacterium infections are more common in men, potentially due to poorer oral hygiene [10]. Given these associations, oral health and dentition should be routinely assessed in patients presenting with brain abscesses, and empiric therapy for anaerobic bacteria should be considered when oral hygiene is poor [11,12].

Before 2011, our patients were treated with oral levofloxacin after hospital discharge, often for more than 12 weeks. This prolonged treatment duration may have been due to a lack of awareness regarding appropriate antibiotic use at the time. Since 2011, antibiotic therapy in our study has generally ranged from six to eight weeks, aligning with the latest European guidelines on brain abscess management. In recent years, our hospital’s Department of Infectious Disease and Infection Control Team has been increasingly involved in patient care, likely improving antibiotic selection. In Japan, no specific guidelines exist for brain abscess treatment, so clinical management often follows bacterial meningitis guidelines, which were introduced around 2010. The shift in antibiotic duration and selection before and after this period may reflect evolving treatment concepts following the establishment of these guidelines.

Surgical treatment of brain abscess

Aspiration and excision are the most commonly performed surgical procedures for brain abscesses. Generally, surgery is indicated when an abscess is ≥2.5 cm in maximum diameter or when antibiotic therapy alone is ineffective [13]. In recent years, aspiration has been recommended as the initial treatment for supratentorial brain abscesses [14]. While aspiration is less invasive than excision, it carries a higher likelihood of requiring reoperation [15].

Sanaullah et al. reported that aspiration was associated with lower costs and fewer postoperative complications, including shorter hospital stays, reduced rates of headache, vomiting, neurological deficits, seizures, and mortality, compared to excision [14]. However, they also noted that aspiration had a higher recurrence rate for multilocular abscesses. Similarly, Chowdhury et al. recommended excision for superficial multilocular brain abscesses [4]. Our study also found that multilocular abscesses were less likely to recur after excision, suggesting that aspiration alone may be insufficient and that excision should be preferred in such cases. Erdoğan and Cansever suggested that excision is indicated for abscesses causing significant mass effect, increased intracranial pressure, diagnostic uncertainty, trauma with foreign material, posterior fossa location, or suspected fungal infection [5].

Dhar and Pal reported that conservative treatment of abscesses <2.5 cm in diameter is significantly associated with a higher recurrence rate and that aspiration yields better outcomes, contradicting previous recommendations for antibiotic therapy alone [16]. Abscess drainage is feasible for lesions >1 cm, and surgical intervention remains the only definitive method for isolating and identifying the causative organism [2,6].

Mortality associated with brain abscesses has declined significantly over time, with recent studies reporting rates below 10%. However, abscess rupture into the ventricular system often leads to ventriculitis and hydrocephalus, conditions linked to mortality rates ranging from 27% to 85% [1,3,17]. IVROBA is a predictor of poor outcomes and high mortality. In our study, the mortality rate among patients with IVROBA was 42.9%, compared to 9.09% in those without IVROBA (p = 0.25, statistical power: 0.37). The overall mortality rate was 22.2%, which is higher than that reported in recent studies. We speculate that this is due to the relatively high incidence of IVROBA (38.8%) in our cohort. Notably, all patients who underwent EVD in this study had IVROBA. EVD should be considered for patients with brain abscesses complicated by hydrocephalus or IVROBA [3]. Additionally, intrathecal antimicrobial administration via a ventricular drainage tube is an effective treatment for IVROBA [18]. However, in the presence of antimicrobial therapy, IVROBA may not necessarily have significant adverse clinical effects, as the abscess is rendered aseptic [18]. Hematogenous brain abscesses, typically located deep at the grey-white matter junction, often have poorly formed capsules, increasing the risk of IVROBA as the abscess enlarges [3,8]. Thus, early surgical intervention combined with medical therapy may be preferable for hematogenous brain abscesses to prevent IVROBA.

Takeshita et al. identified localized enhancement of the ventricular wall adjacent to an abscess as a risk factor for IVROBA [19]. In our study, several IVROBA cases involved abscesses in direct contact with the ventricles, with contrast enhancement visible on the ventricular walls. However, these findings were only observed after ventricular perforation, highlighting the need for early prediction and intervention. Lee et al. reported that multiloculated brain abscesses are 4.2 times more likely to cause IVROBA than simple abscesses and that a reduction of just 1 mm in the distance between the abscess and the ventricular wall increases the rupture risk by 10% [20]. Therefore, excision may be a more appropriate treatment for multiloculated brain abscesses located near the ventricles to minimize the risk of IVROBA.

In our study, two patients experienced postoperative cerebral hemorrhage, both of whom were discharged with an mRS score of 6. One patient developed a hemorrhage along the aspiration tract, while the other had an incidental brainstem hemorrhage at a site unrelated to the surgery. Helweg-Larsen et al. reported that postoperative bleeding is the most common complication following brain abscess surgery, though fatal cases are rare [17]. One of our patients with tract bleeding had chronic myelogenous leukemia and was receiving platelet transfusions, which may have contributed to an increased risk of bleeding complications.

Recurrence of brain abscess

Reported recurrence rates for brain abscesses range from 10% to 50%, highlighting the need for at least one year of follow-up after treatment. [5] In our study, the overall recurrence rate per procedure was 18.2%, with all cases requiring reoperation. However, neurosurgeons should remain cautious of the risks associated with repeated surgeries. Chowdhury et al. reported that repeat aspiration for recurrent cerebellar abscesses is linked to neurological deterioration, suggesting that excision may be the preferred approach in such cases [4].

In three cases of recurrence in our study, the initial treatment was aspiration with or without drain placement, followed by frequent postoperative non-contrast CT monitoring. It is likely that non-contrast CT was insufficient to reliably detect recurrent abscesses. Multiple studies have underscored the importance of appropriately timed imaging to assess treatment efficacy [21,22]. In retrospect, contrast-enhanced MRI would have been more effective in confirming recurrence. In our study, contrast-enhanced MRI was typically performed about one week after surgery, with the decision to reoperate left to the treating surgeon. Notably, the diameter of recurrent abscesses did not differ significantly from their preoperative size, suggesting that multilocular abscesses may have been challenging to fully drain and were therefore incompletely treated.

Although few studies have specifically analyzed clinical outcomes in patients with recurrent brain abscesses, overall prognosis has improved due to advancements in diagnostic imaging, antimicrobial therapy, minimally invasive neurosurgical techniques, and improved bacterial culture methods [1].

Limitations

The primary limitation of this study is its retrospective, single-center design, which may have introduced selection bias. Additionally, the small sample size could lead to inadequate statistical power. Since the study was conducted at a single institution, the generalizability of the findings may also be limited.

Moreover, the approach to abscess treatment varied among surgeons and was not standardized over the 17-year study period, potentially resulting in inconsistencies in management. The involvement of multiple surgeons with differing techniques further contributed to the lack of uniformity in treatment.

## Conclusions

Surgical treatment of brain abscesses should take into account both the multilocular nature of the abscess and the status of the primary infection. Aspiration for multilocular brain abscesses may be associated with a higher recurrence rate. Further case accumulation is needed to confirm whether excision should be the primary treatment for multilocular brain abscess.

## Appendices

**Table 5:** Patient table ^* ^6, 12: twice operated due to recurrence; 15: three times operated due to two recurrences ^†^ 8, 11: two cases detected two different types of bacteria EVD, external ventricular drainage; IVROBA, intraventricular rupture of brain abscess; mRS, modified Rankin scale

No.	Age	Sex	Location	Cyst type	Initial surgical procedure^*^	IVROBA	Pre-onset mRS	mRS at Discharge	Recurrence	Pathogenic bacteria^†^	Mean long diameter (cm)	Mean volume (cm³)	Notes
1	76	M	Temporal, R	Simple	Aspiration	+	3	3	-	α-Hemolytic *Streptococcus*	No data	No data	-
2	31	M	Occipital, L	Simple	Excision	-	0	0	-	*Haemophilus* spp.	No data	No data	-
3	75	M	Frontal, L	Simple	Aspiration	-	4	4	-	*Streptococcus* epidermidis	No data	No data	-
4	39	M	Frontal, R	Simple	Aspiration	-	0	1	-	α-Hemolytic *Streptococcus*	5.3	33.28	-
5	45	M	Frontal, R	Simple	Aspiration	-	2	6	-	-	2.7	3.39	Leukemia, postoperative cerebral hemorrhage
6	72	M	Frontal, R	Simple	Aspiration + EVD	+	3	6	+	Staphylococcus aureus	4.2	17.14	Lung cancer
7	78	M	Frontal, R	Simple	Aspiration	-	0	3	-	-	4.4	26.6	-
8	79	F	Cerebellum, L	Simple	Aspiration	-	3	5	-	*Corynebacterium *species, *Enterococcus faecalis*	3.6	11.3	Upper eyelid Merkel cell tumor, post-irradiation petrosal bone infection
9	62	M	Frontal, R	Simple	EVD	+	0	3	-	α-Hemolytic *Streptococcus*	4.8	36.17	Esophageal cancer
10	52	M	Thalamus, R	Simple	Aspiration	-	0	0	-	α-Hemolytic *Streptococcus*	2.2	4.61	-
11	73	M	Frontal, R	Simple	EVD	-	1	5	-	*Fusobacterium* species, *Peptostreptococcus *species	2	1.63	-
12	66	M	Occipital, R	Multi	Aspiration (repeated)	-	0	1	+	*Peptostreptococcus* micros	2.6	4.08	-
13	79	M	Frontal, R	Simple	Aspiration	-	0	0	-	-	2.1	2.64	Bedsore
14	88	M	Frontal, L	Simple	EVD	+	0	6	-	*Streptococcus milleri *group	3.7	14.52	Postoperative brainstem hemorrhage
15	64	M	Parietal, R	Multi	Aspiration + excision	-	2	3	+	-	4.3	44.13	Leg intramuscular abscess
16	84	M	Frontal, R	Simple	EVD	+	3	6	-	Streptococcus intermedius	1.6	2.46	-
17	66	M	Frontal, L	Simple	Aspiration	-	0	3	-	Porphyromonas asaccharolytica	5.9	48.17	Stomach cancer, sinusitis
18	75	M	Cerebellum, L	Simple	EVD + aspiration	+	2	5	-	Fusobacterium nucleatum	2.3	4.33	-
